# A correlation between Magnetic Resonance Spectroscopy (1-H MRS) and the neurodevelopment of two-year-olds born preterm in an EPIRMEX cohort study

**DOI:** 10.3389/fped.2022.936130

**Published:** 2022-08-19

**Authors:** Catherine Gire, Julie Berbis, Marion Dequin, Stéphane Marret, Jean-Baptiste Muller, Elie Saliba, Barthélémy Tosello

**Affiliations:** ^1^Department of Neonatal Medicine, Assistance Publique Hopitaux de Marseille, Marseille, France; ^2^EA3279, Faculty of Medicine, Self-Perceived Health Assessment Research Unit, Marseille, France; ^3^Department of Neonatal Pediatrics, Intensive Care, and Neuropediatrics, Rouen University Hospital and Institut National de la Santé et de la Recherche Médicale INSERM U 1245 Team 4 Neovasc, School of Medicine, Normandy University, Rouen, France; ^4^Department of Neonatal Medicine, Nantes University Hospital, Nantes, France; ^5^UMR 1253, iBrain, Tours University, Institut National de la Santé et de la Recherche Médicale (INSERM), Tours, France; ^6^Aix-Marseille University, CNRS, EFS, ADES, Marseille, France

**Keywords:** neurodevelopmental outcome, magnetic resonance spectroscopy, very preterm infant, morbidity, 2 years follow-up

## Abstract

**Background:**

Preterm infants are at risk of neurodevelopmental impairments. At present, proton magnetic resonance spectroscopy (1H-MRS) is currently used to evaluate brain metabolites in asphyxiated term infants. The purpose of this study was to identify in the preterm EPIRMEX cohort any correlations between (1H-MRS) metabolites ratio at term equivalent age (TEA) and neurodevelopmental outcomes at 2 years.

**Methods:**

Our study included EPIRMEX eligible patients who were very preterm infants (gestational age at birth ≤32 weeks) and who underwent a brain MRI at TEA and ^1^H-MRS using a monovoxel technique. The volumes of interest (VOI) were periventricular white matter posterior area and basal ganglia. The ratio of N Acetyl Aspartate (NAA) to Cho (Choline), NAA to Cr (creatine), Cho to Cr, and Lac (Lactate) to Cr were measured. Neurodevelopment was assessed at 24 months TEA with ASQ (Ages and Stages Questionnaire).

**Results:**

A total of 69 very preterm infants had a 1H-MRS at TEA. In white matter there was a significant correlation between a reduction in the NAA/Cho ratio and a total ASQ and/or abnormal communication score, and an increase in the Lac/Cr ratio and an abnormality of fine motor skills. In the gray nuclei there was a trend correlation between the reduction in the NAA/Cho ratio and sociability disorders; and the increase in the Lac/Cr ratio and an anomaly in problem-solving.

**Conclusions:**

Using NAA as a biomarker, the vulnerability of immature oligodendrocytes in preterm children at TEA was correlated to neurodevelopment at 2 years. Similarly, the presence of lactate at TEA was associated with abnormal neurodevelopment at 2 years in the preterm brain.

## Introduction

Preterm birth mortality rates have decreased as neonatal care has advanced.

However, because of numerous associated medical factors, preterm survivors remain at high risk for neurodevelopmental impairments (NDIs) (1) such as cerebral palsy (CP), as well as neurocognitive, behavioral, and motor impairments. Nearly 40% of very preterm infants experienced increased NDIs (2) with consequences on social skills and academic underachievement later in life (3).

Thus, a prediction of NDIs because of prematurity is crucial for proper clinical assessment, parental counseling, managing neurodevelopmental follow-up, and the development of future neuroprotective strategies.

Cerebral magnetic resonance imaging (MRI) at term equivalent age (TEA) is valuable to track brain development because of the data resolution obtained and its non-invasive nature. Conventional sequences detailing cerebral morphology have helped to define encephalopathy in preterm infants (4, 5). There are numerous studies showing an association between white matter abnormalities and sensorimotor disorders such as periventricular leukomalacia and cerebral palsy. However, acute and chronic injury at a cellular level is sometimes difficult to identify on anatomical neuroimaging. Infants without any abnormalities seen on conventional MRIs may later present neurodevelopment and/or behavioral impairments. A more accurate prediction of neurodevelopmental outcomes is therefore important for clinical management (1, 6).

Magnetic resonance spectroscopy (1H-MRS) at TEA may provide additional information in combination with conventional MRIs performed on infants. 1H-MRS can non-invasively measure various brain metabolites that are known to be altered during rapid brain development in the first year of life (7). It provides a qualitative and quantitative analysis of several metabolites participating in the cerebral cellular energy cycle by using the magnetic properties of certain atoms. Studies using 1H-MRS have reported rapidly changing concentrations of metabolites, particularly in the first few months of life as related to developmental processes, and may be an important method to improve the assessment of neonatal brain development and injury (8).

Previous 1H-MRS studies have demonstrated changes in white matter metabolism with brain development (9–12). These changes included an increase in N Acetyl Aspartate (NAA) and decreases in both Choline (Cho) and Lactate with progressing brain maturity. NAA is an amino acid synthesized primarily in neurons and/or axonal mitochondria (13). NAA plays a part in myelination, metabolism of brain fatty acids, ion balance, and neuromodulation. Its presence is detectable in the white matter and cortex as early as 16 weeks GA (Gestation Age) and increases during pre- and post-natal brain development, thus becoming the dominant 1H-MRS peak at approximately 6 months of TEA. Afterward, NAA, a marker for neuronal activity, increases gradually beginning at 24 weeks GA as the brain matures. Concurrently, Cho, a marker for membrane turnover and myelination, decreases with age as the rapid brain growth of the neonate begins to slow in infancy. The 1H-MRS' Cho peak includes free Choline, phosphocholine, and glycerophosphocholine. Creatine reflects energy metabolism and has been shown to increase postnatally and just before term (8).

We hypothesized that 1H-MRS can be used at TEA as a potential tool to predict neurodevelopmental outcomes in preterm infants in (this) population. By using this approach, our goal was to identify in the large EPIRMEX cohort nested in a population-based epidemiological survey (EPIPAGE 2) the correlations between the 1H-MRS ratio at TEA and the neurodevelopmental outcomes at 2 years.

## Methods

### Participants

Between 28 June 2011 and 31 October 2012, we recruited 519 preterm infants ranging from 26–31 completed weeks GA. This recruitment, from a national perspective longitudinal cohort study, came from 16 university hospital centers in 12 regions in France. Each infant had an MRI at TEA: EPIRMEX ancillary cohort study associated with EPIPAGE 2 (Etude Epidémiologique sur les Petits Âges Gestationnels-2). The present research project therefore deliberately focuses on brain imaging at term (39–41 GA), as well as on neurodevelopmental assessment. These two specific aspects are covered respectively by EPIRMEX and EPIPAGE 2. Indeed, our analysis will focus on a sub-sample of children born prematurely included in the national cohort EPIPAGE 2 and included in EPIRMEX (ancillary study of EPIPAGE-2 on a sub-sample of children born between 26 GA+0 d and 32 GA+6) and having benefited from a brain MRI with spectroscopy. These children underwent conventional MRI at term (39–41 GA), completed by specific sequences for microstructural analysis in monovoxel analysis (white matter and gray nuclei), short echo, and long echo.

### Inclusion criteria

These criteria consisted of all live births occurring between 22 and 31 GAs that also received an MRI at TEA. The follow-up population included all children with a live discharge from either a neonatal intensive care, special unit, or maternity ward. Each child had parental approval to participate in the study (14).

### Non-inclusion criteria

**Figure 1:**
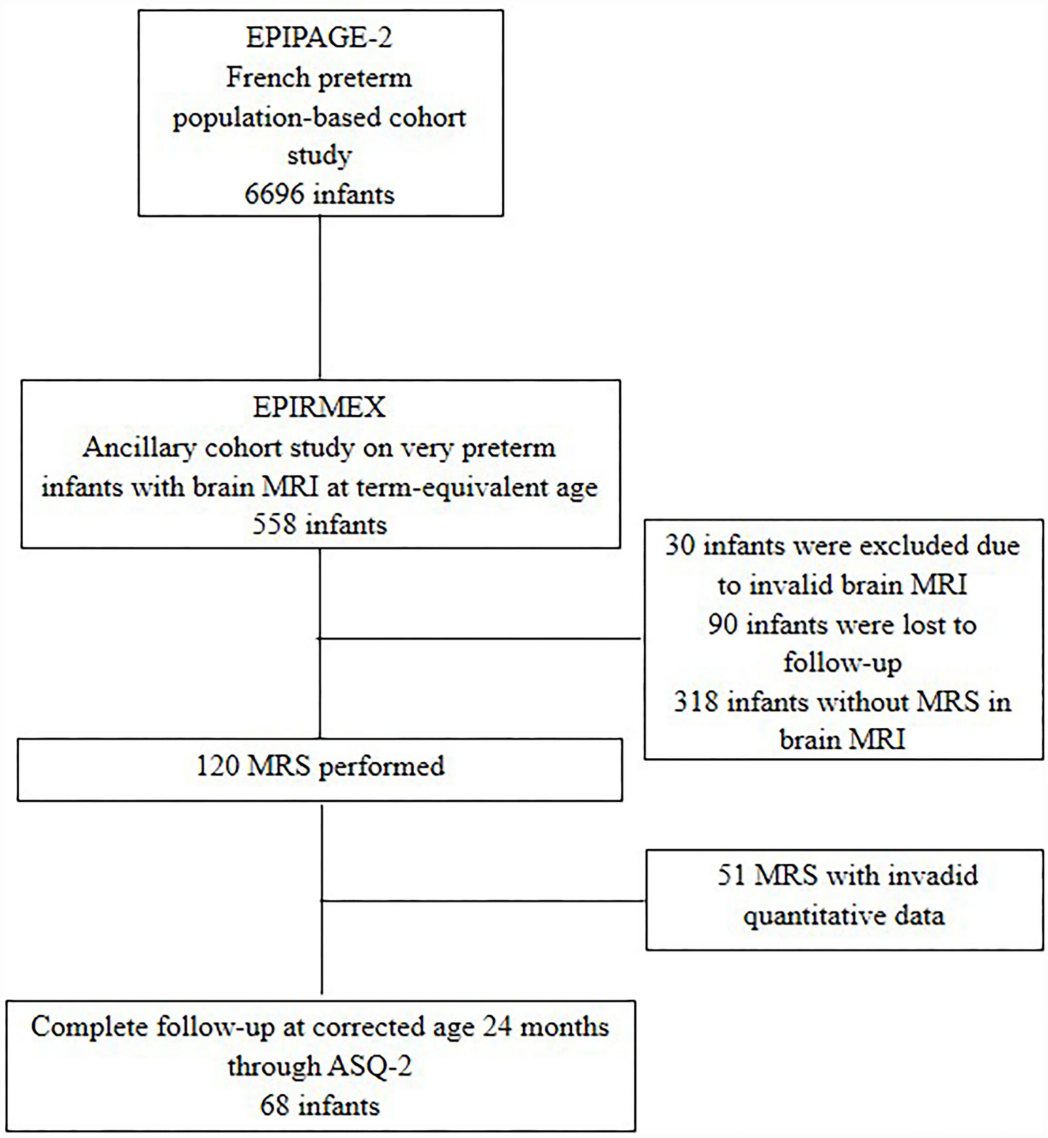
Study flow-chart.

These criteria were children with severe karyotypic abnormalities, and ante-natal severe central nervous system malformations, or whose parents' declined participation. Also excluded were 99 patients with incomplete clinical data as well as 30 infants having had brain MRIs at TEA with severe artifacts (non-interpretable). Among these MRIs, 110 1H-MRS were performed and 69 metabolite ratios could be extracted (Figure 1: Flow chart).

### Ethical statement

Data collection and evaluation for this study were approved by the Committee for the Protection of Persons (CPP-Ouest-1, 10/03/2013, ref 2011 R19) and written consent was obtained from all participating families.

### Study design

#### MRI/MRS protocols

MRIs were done without sedation during the infants' natural sleep. Infants were positioned inside the scanner wrapped in a vacuum pillow and monitored with electrocardiography and pulse oximetry. Earmuffs were used to minimize noise reduction.

MRI brain scans were performed using a 1.5T or 3T MRI system with a dedicated 8-channel head coil. The MR devices with a magnetic field of 1.5T were Philips Achieva^®^, Philips Intera^®^, Toshiba MRT 200^®^, GE SignaHdxt^®^ (General Electric Healthcare), Siemens Avanto^®^, Siemens Symphony^®^, and Siemens SymphonyTim^®^ (Siemens Heathineers).

The MRI device with a 3T magnetic field was the Philips Achieva^®^ (Philips Healthcare). We obtained T2 datasets by using an axial T2 morphological sequence (fast spin echo/turbo spin echo with a 90 flip-back pulse); slice thickness, 3 mm; pixel size, 0.39 × 0.39 mm2; field of view, 192 mm; repetition time, 6680 ms; echo time, 142 ms; and flip angle, 120. The axial MRI reference plane was the bi-commissural plane. MRI protocol also included an axial T2^*^-weighted sequence, an axial diffusion-weighted sequence (b = 1,000 s/mm^2^), and a three-dimensional T1 MPRage sequence (spatial resolution, 0.8 × 0.8 × 1.2 mm^3^; inversion time, 110 ms; repetition time 2,200 ms; echo time, 2.49 ms; flip angle, 8°) (14).

#### Brain MRI data

The total maturation score is calculated by taking the sum of the myelination score (1–7), cortical infolding score (1–6), germinal matrix score (1–4), and bands of migrating glial cells score (l1–l4). The higher the score, the more advanced the maturation (6).

The use of a standardized scoring system (Appendix 1) made it possible to semi-quantitatively evaluate the presence of lesions and their severity in both the white and gray matter and the cerebellum. The cerebral white matter scale (CWM) is the sum of six subscales:

(1) Presence and severity of cystic lesions,

(2) Signal abnormality,

(3) Thinning of the corpus callosum,

(4) Lateral ventricular dilatation,

(5) Periventricular white matter loss, and

(6) Punctate lesions.

Each item was rated from 0 to 3, except for punctate lesions which were rated from 0 to 2. The CWM score was considered normal if ≤5; slightly abnormal if between 6–11; and moderately to severely abnormal if between 12–17 (4). This was then grouped into two categories (normal and slightly abnormal/moderately to severely abnormal).

The cortical gray matter scale (CGM) was the sum of three subscales, with a score of 0 to 3 (15).

(1) Signal abnormality,

(2) Delayed gyral maturation, and

(3) Increased extra cerebral space.

The CGM scale was normal if ≤3, slightly abnormal if the score was between 4–6, and moderately to severely abnormal with a score between 7–9.

We grouped these into two categories (normal and slightly abnormal/moderately to severely abnormal).

The cerebellum scale was considered normal in the absence of any hemorrhagic punctate abnormality, slightly abnormal if the bleeding lesion was unilateral, and moderately to severely abnormal if the abnormalities were bilateral, multiple, or associated with a reduction in the volume of the cerebellum.

Four measurements were added to the analysis (6, 16). The measurements were taken from the coronal section and manually calculated:

The diameter of the lateral ventricles in order to assess ventricular dilatation (VD)
The biparietal diameter (BPD) to evaluate any brain volume reduction
The interhemispheric distance (IHD) to study the extra cerebral space
The trans-cerebellum diameter (TCD) to study cerebellum volume.

#### 1H-MRS data

This study used a monovoxel technique to obtain spectroscopy. Indeed, the metabolic profile in premature babies at TEA must correspond to cerebral maturation and therefore to cerebral vulnerability. There is a diffuse white matter anomaly in almost 50% of very preterm infants (17). In this metabolic study, the voxel was placed in two regions of interest: the posterior periventricular white matter region, and basal ganglia and thalamus by convention on the right side. The myelination process is well known with a very specific order from caudal (spinal, brainstem, and basal ganglia) to the rostral (telencephalic) (8). The posterior periventricular white matter in the immature brain is particularly vulnerable due to the susceptibility of preoligodendrocytes to hypoxia-ischemia and/or inflammation. At the same time, an axonal lesion of the white matter, and/or due to premature birth, can disturb the reciprocal connection of the thalamus with the entirety of the cerebral cortex *via* the axons (18). A decrease in thalamic volume in premature babies is associated with abnormal metabolism of the parietal white matter independent of the lesion (10). A loss of thalamic volume with white matter microstructural modifications in premature babies at TEA is associated with neurodevelopmental disorders (19). Furthermore, thalamocortical connectivity in preterm infants, corrected by diffusion MRI, is correlated with cognitive development at 2 years of age (20). Metabolic alteration in the thalamus thus results from delayed maturation of thalamocortical systems and/or neuronal loss due to subtle axonal lesions of white matter.

The size of the nominal volume of interest (VOI) is 4.5 cm3 (20 ^*^ 15 ^*^ 15 mm^3^) as recommended. This volume represents the minimum acceptable size to obtain the spectra with sufficient signal-to-noise ratio (21). Long echo sequences were performed which are characterized by a repetition time (TR) of 1,500 ms, a time to echo (TE) of 135 ms, and 192 acquisitions with an acquisition time of 3 min,14 s to collect the NAA signals of Cr, of Cho, of Cr and Lac. The peak intensity of metabolites is age dependent and displays regional variations. NAA/Cho significantly increased with age for all the regions of interest across the entire premature newborn brain (22). There were higher NAA and Cho peaks in the parieto-occipital areas of the white matter than in the frontal areas, indicating better myelination in this area (23). Cerebral maturation was reflected by an increase in NAA (a marker of oligodendrocyte proliferation and differentiation), Cr with a concomitant decrease in Cho. Lactate peaks found in premature babies are not regarded as normal in typical white matter maturation. Peaks of Cho, Cr, and NAA occur first in the basal ganglia and thalami in premature infants thus reflecting strong proliferative activity in these areas as compared to the white matter. The concentration of NAA is higher in the gray matter than in the white matter (expressed by the mitochondria of the cell body and not in the axon or the prolongation of the oligodendrocytes). Creatine is also higher in the gray matter than white matter and is not found in mature oligodendrocytes. Choline is a precursor to acetylcholine which is the major brainstem neurotransmitter responsible for signaling pathways. Choline levels increase with cell proliferation, membrane turnover, myelination, or inflammation and decrease with age as rapid brain growth in the neonatal period decelerates (8, 24).

Data were processed using proprietary software developed on IDL (Interactive Data Language, Research System Inc., Boulder, CO). Spectral processing and spectral reconstruction were performed using the automated shimming program provided by the manufacturer after an additional water suppression using the HLSVD-MRUI FORTRAN code. Spectra were fitted using the AMARES-MRUI FORTRAN code (17) by CEREMM in Marseille (25, 26).

### Data collection

To determine gestational age, we based our best estimate from the last menstrual period date and an early prenatal ultrasonogram.

The antenatal and obstetrical data collected at birth were: Complete antenatal corticosteroid therapy (two injections of corticosteroids, 24 h apart). Classification of prematurity related to five causes: preterm labor, preterm premature rupture of membranes, hypertensive disorders without suspected fetal growth restriction (FGR), hypertensive disorders with suspected FGR, and placental abruption after an uncomplicated pregnancy, suspected FGR without hypertensive disorders.

Neonatal morbidities: Intraventricular Hemorrhage (IVH), Cystic Periventricular Leukomalacia stages II or III (cPVL), Necrotizing Enterocolitis (NEC) stage 3 or greater, Retinopathy of prematurity (ROP), stage 3 and/or laser treatment. Severe bronchopulmonary dysplasia (BPD) is defined as requiring oxygen for at least 28 days in addition to the requirement of 30% or more oxygen and/or mechanical ventilator support or continuous positive airway pressure at 36 weeks postmenstrual age

We defined neonatal morbidity criteria as the presence of IVH 3 and/or IVH4 and/or LMPV and/or BPD and/or ROP ≥3 and/or NEC ≥2 (2).

Family socioeconomic status was recorded according to the national French classification of occupations and social position (https://www.insee.fr/fr/information/2406153) and grouped into five categories: (1) professional, (2) intermediate, (3) administrative/public service, self-employed or student, (4) shop assistant, service worker, and (5) manual worker or unemployed. Socioeconomic status was defined as the higher occupation between the two parents or the mother's occupation if she lived alone.

### Developmental assessments at 24 months, correct age

Cerebral palsy was defined according to the criteria of the European Surveillance of Cerebral Palsy in Europe (SCPE) network. Auditory and visual impairments were either unilateral or bilateral. The psychomotor development of those children who were free from cerebral palsy or sensory deficit was assessed using the 24-month Ages and Stages Questionnaire-2 (ASQ-2). A pathological ASQ score was defined as a score of <2 standard deviations in one of the five evaluated domains (2).

### Statistical analysis

A preterm descriptive analysis was performed with qualitative variables being presented as numbers and percentages. The description of demographic, clinical, MRI, and 1H-MRS characteristics, between the follow-up and non-follow-up, was compared using the Mann-Whitney for continuous variables and the X2 test for categorical variables.

We used the Mann-Whitney test to compare perinatal characteristics, MRI abnormalities, and the 1H-MRS ratio of NAA/Cho, NAA /Cr, Cho/Cr, and Lac/Cr in each area of interest (basal ganglia or white matter).

Univariate logistic regression analysis was performed to test the relationship of metabolite 1H-MRS ratios in the basal ganglia or white matter areas of interest, for TEA MRI, with the neurodevelopmental at 24 months. As well as using the multivariate linear regression analysis for the correlation of 1H-MRS's metabolite ratios for the neurodevelopmental at 24 months in order to assess the effect of a given 1H-MRS's metabolite ratio on neurodevelopment independently of term MRI abnormalities, GA, and socioeconomic data.

The statistical analysis was carried out using the R software. All tests were two-sided. The significance threshold was set at 5%.

## Results

### Population

In total, 69 preterm infants were included in this study with complete clinical data, MRI, and a 1H-MRS metabolite ratio (Figure 1). The mean GA was 27.9 (+/−1.6) weeks GA, and the birth weight was 1,067 (+/−259.5) g. The maternal-obstetrical and neonatal characteristics are presented in Table 1.

**Table 1:** Characteristics of the study population.

	***N* (%)**
***Maternal and obstetrical characteristics*** **(*****N*****: 69)**	
Maternal age (years. mean +/–SD)	29.2 (2.1)
Family socio economic status	
Professional	13 (18.8)
Intermediate	6 (8.7)
Administrative/public service. self–employed or student	13 (18.8)
Shop assistant. Service worker	10 (14.5)
Manual worker or unemployed	12 (17.3)
Maternal smoker	15 (21.7)
Complete ACS	40 (57.9)
Cesarean section	51 (73.9)
***Postnatal characteristics***	
GA (weeks mean +/–SD)	27.9 (1.6)
GA 24^0/7^-27^6/7^ weeks	27 (39.1)
GA 28^0/7^-31^6/7^ weeks	42 (60.9)
Delayed cord clamping	14 (20.3)
Male n (%)	42 (60.9)
Birth weight, g mean (+/–SD)	1,067 (259.5)
Birth weight <3°p	32 (46.4)
Birth weight <10°p	38 (55.1)
Apgar <7	4 (5.8)
Tracheal intubation	41 (59.4)
Chest compression	8 (11.6)
Severe neonatal morbidity*	27 (39.1)
BPD	26 (37.7)
ROP	1 (1.4)
IVH 1–2	20 (28.9)
IVH 3–4	1 (1.4)
cPVL	3 (4.3)
***Brain MRI***	***N** **=*** **67**
Cerebral maturation score: (*n =* 67)	12.88 [SD = 1.78, IC (10–19)]
Abnormal WM scale (*n =* 67)	39 (58)
Abnormal GM scale (*n =* 67)	16 (23)
DESHI (diffuse excessive high signal intensity) (yes) (*n* = 67)	44 (65*)*
Lateral ventricular dilation (*n =* 64)	14 (22)
Reduction of Trans Cerebellum Diameter (*n =* 64)	21 (32)
Reduction of WM volume (*n =* 64)	32 (50)
Increased extra–cerebral space (*n =* 64)	42 (66)
Deep GM volume reduction (*n =* 63)	3 (4.8)

ACS, antenatal corticosteroid therapy; GA, gestational age; BPD, Broncho pulmonary dysplasia; ROP: retinopathy of prematurity; IVH: intraventricular hemorrhage; cPVL: cystic periventricular leukomalacia; GM: gray matter; WM: white matter.

^*^Severe neonatal morbidity (defined by IVH ≥ grade 3 and/or cPVL and/or BPD and/or ROP ≥ stage 3 and/or NEC ≥ stage 2).

Quantitative variables are expressed as means ± standard deviations; numbers in brackets are ranges. Qualitative variables are expressed as raw numbers; numbers in parentheses are proportions followed by percentages. Bold indicates a significant P–value.

**Data Brain MRI**

Total maturation score: sum of Myelination Score (1–7), Cortical infolding Score (1–6), Germinal Matrix Score (1–4), Bands of migrating glial cells Score (l1–l4). The higher the score, the more advanced the maturation.

The use of a standardized scoring system made it possible to semi–quantitatively evaluate the presence of lesions and their severity in the white and gray matter, as well as in the cerebellum.

The cerebral white matter scale (CWM) is the sum of six subscales:

(1) Presence and severity of cystic lesions,

(2) Signal abnormality,

(3) Thinning of the corpus callosum,

(4) Lateral ventricular dilation,

(5) Periventricular white matter loss, and

(6) Punctate lesions.

Each item was rated from 0 to 3, except for punctate lesions which were rated from 0 to 2. The CWM score was considered normal if ≤5, slightly abnormal if it was between 6–11, and moderately to severely abnormal if it was between 12–17 (4). We have grouped them into two categories: normal ≤5 and abnormal > to 5.

The cortical gray matter scale (CGM) was the sum of three subscales. with a score of 0 to 3 (15):

(1) Signal abnormality,

(2) Delayed gyral maturation, and

(3) Increased extra cerebral space.

The CGM scale was normal if ≤3, slightly abnormal if the score was between 4–6, and moderately to severely abnormal if between 7–9. We have grouped them into two categories: normal ≤3 and abnormal >or equal to 3

The cerebellum scale was considered normal in the absence of any hemorrhagic punctate abnormality, slightly abnormal if the bleeding lesion was unilateral, and moderately to severely abnormal if the abnormalities were bilateral, multiple, or associated with a reduction in the volume of the cerebellum. We have grouped them into two categories: normal or abnormal (slightly abnormal, moderately to severely abnormal).

Four measurements were added to the analysis (16). The measurements were taken from the coronal section and manually calculated.

By using these measures, patterns of impaired brain growth were identified (16):

1. Biparietal corrected diameter: no anomaly ≥ 77 mm; mild anomaly: ≥72 mm and <77 mm; moderate to severe anomaly <72 mm.

2. Corrected trans–cerebellum diameter: no anomaly: >47 mm; moderate to severe anomaly: ≤ 47 mm.

3. Ventricular dilation: normal or mild anomaly (Bilateral <7.5 mm or unilateral ≤ 10 mm); moderate to severe anomaly (Bilateral or unilateral ≥ 10 mm).

4. Interhemispheric distance: normal or mild anomaly (<5 mm); moderate to severe abnormality (≥ 5 mm).

In half of the cases, we found that the MRI lesions were white matter abnormalities (WMA) in 39/67 (58%) and diffuse excessive high signal intensity (DESHI) in 45/69 (66%) of the cases. An anomaly of white matter volume and the cerebellum were found respectively in 50% (32/69) and 33% (21/69) of the cases.

The ASQ neurodevelopmental outcomes were assessed at 24 months of age in 60 of the 69 children (85.5%). In our cohort, an ASQ score below the threshold was observed in 37% (22) cases. The score most frequently found below the threshold score was in the communication domain (36%) (Table 2).

**Table 2:** Results of the 24–month ages and stages questionnaire−2.

***ASQ assessment***	***N =* 60**
Age, months (median [IQR])	26.5 [25.7–29]
Term equivalent age, months (median, IC)	24 [23–25.2]
Score total ASQ [mean (+/SD); median (IC)]	231.2 (61.3); 245 [195–280]
Abnormal ASQ (*n =* 59)	22 (37.5.)
Abnormal Communication (*n =* 60)	22 (36.6)
Abnormal Gross Motor (n = 59)	7 (11.9)
Abnormal Fine Motor (*n =* 60)	6 (10.1)
Abnormal Problem Solving (*n =* 60)	6 (10.3)
Abnormal Personal and Social Skills (*n =* 60)	11 (18.3)

ASQ, The psychomotor development of those children free from cerebral palsy or sensory deficit was assessed using the 24–month Ages and Stages Questionnaire (ASQ). The ASQ was validated in France and completed by parents. A pathological ASQ score was defined as a score of <2 standard deviations in one of the five domains evaluated.

For those children with or without receiving an ASQ assessment at 2 years of age, we found no differences in MRI abnormalities at TEA, nor any 1H-MRS metabolite ratio differences. One exception, however, was a reduction in white matter volume on cerebral MRI in those children not followed up at 2 years We also found a trend toward an increase in the NAA/Cho ratio in WM (*p* = 0.068) (Table 3).

**Table 3:** Children's ASQ follow–up vs. non–follow–up.

	**1–HMRS with ASQ (*n =* 59), *N* (%)**	**1–HMRS without ASQ (*n =* 10), *N* (%)**	***p*–value**
Age of mother	30.95 (6.2)	28.75 (7.7)	0.30
Family SES	48/60 (81)	6/10 (60)	0.61
ACS	48/60 (81)	7/10 (70)	0.6
Weight <1,000 g	26/59 (44)	4/10 (40)	1
Male	36/59 (61)	6/10 (60)	1
GA weeks (mean +/–SD)	28.1 (1.7)	27.6 (1.3)	0.38
GA 24^0/7^-27^6/7^ weeks	21/59 (36)	6/10 (60)	0.28
GA 28^0/7^-31^6/7^ weeks	38/59 (64)	4/10 (40)	
**Brain MRI abnormalities**			
WM injury score abnormal	33/57 (57)	6/10 (60)	0.73
GM injury score abnormal	16/57 (28)	0/10 (0)	0.1
DESHI	37/57 (35)	7/10 (30)	1
LVD	23/54 (42)	1/10 (10)	0.23
TCD	16/54 (29)	5/10 (50)	0.30
BPD	23/54 (42)	9/10 (90)	0.018
IHD	37/54 (68)	5/10 (50)	0.64
**Metabolite peak area ratios (Time echo 135 ms)**			
NAA/Cho (in WM)	0.68 (0.14)	0.75 (0.10)	0.068
NAA/Cr (in WM)	1.65 (0.75)	1.29 (0.2)	0.25
CHO/Cr (in WM)	2.41 (0.97)	1.73 (0.16)	0.015
NAA/Cho (in GM)	0.74 (0.16)	0,73 (0.11)	0.99
NAA/Cr (in GM)	1.28 (0.32)	1.22 (0.13)	0.73
CHO/Cr (in GM)	1.79 (0.52)	1.7 (0.2.6)	0.85

SES, socio–economic status; ACS, antenatal corticosteroid therapy; GA, gestational age; WM, white matter; GM, gray matter; DEHSI, diffuse excessive high signal intensity; TCD, Trans Cerebellum Diameter (moderate to severe anomaly, ≤47 mm); BPD, biparietal diameter (moderate to severe anomaly <72 mm); IHD, inter hemispheric diameter [Increased Extracerebral Space, moderate to severe abnormality (≥5 mm)], NAA, N Acetyl Aspartate; Cr, Creatinine; Cho, choline; Lac, lactate.

### H1-MRS ratio, perinatal data, and MRI

All the results of the NAA/Cho, NAA/Cr, Cho/Cr, and Lac/Cho ratios are presented in Table 4. In gray and white matter, the values of each 1H-MRS ratio were close to each other.

**Table 4:** Metabolite peak area ratios at term–equivalent age (TEA).

	**Gray matter**				**White matter**			
	**NAA/Cho**	**NAA/Cr**	**Cho/Cr**	**Lac/Cr**	**NAA/Cho**	**NAA/Cr**	**Cho/Cr**	**Lac/Cr**
N	59	59	59	59	63	63	63	63
Data missing	10	10	10	10	6	6	6	6
Mean	0.74	1.28	1.78	0.11	0.69	1.60	2.31	0.19
Median	0.74	1.24	1.66	0.00	0.67	1.38	2.02	0.00
SD	0.15	0.30	0.49	0.18	0.13	0.71	0.93	0.28
Min	0.324	0.56	0.97	0.00	0.34	0.586	1.01	0.00
Maxi	1.490	2.16	3.44	0.76	1.02	3.65	5.16	1.39
Cent 25	0.68	1.04	1.45	0.00	0.62	1.17	1.67	0.00
Cent 75	0.79	1.41	1.97	0.20	0.75	1.79	2.47	0.34

SD, standard deviation; NAA, N Acetyl Aspartate, Cr, Creatine, Cho, choline, Lac, lactate.

Data are shown as medians and interquartile range.

TEA, Period between 37 Weeks GA and 41 Weeks GA.

Locations of the measurement's voxels were periventricular posterior area at the level of the white matter (WM) and the thalamus and basal ganglia, volume of interest (VOI). Long echo times were always in the right panel.

There were no significant associations between antenatal corticosteroid therapy, magnesium sulfate, weight, GA, and the ratios of 1H-MRS in either the gray or the white matter.

In the gray matter, there was a difference but not significant between (1) weight ≤ 1000 g and an increase in the mean of the NAA/Cr (*p* = 0.08) and Cho/Cr (*p* = 0.09) ratios; (2) an abnormality in the volume of the white matter on MRI and an increase in the mean Lac/Cr ratio (*p* = 0.05); and (3) an increase NAA/ Cho and an inverse relationship to the maturation score (OR: 0.04, *p* = 0.02).

In the white matter, there was a statistically significant link between the existence of DESHI and the decrease in the mean NAA/Cho ratio (*p* = 0.02), the inverse maturation score, and the increase in the mean NAA/Cho ratio (OR: 0.4, *p* = 0.02). There was a difference but not significant between (1) the existence of DESHI and the increase in the mean Cho/Cr ratio (*p* = 0.05); (2) the presence of ventricular dilatation and the decrease in the mean NAA/Cho ratio (*p* = 0.05) and the increase in the Cho/ Cr ratio (*p* = 0.14); and (3) an abnormal diameter of basal ganglia and a decrease in the mean NAA/Cho ratio (*p* = 0.15) and an increase in the mean Lac/Cr ratio (*p* = 0.14) (Table 5).

**Table 5:** Correlation between mean metabolite peak area ratios in gray matter **(A)** and white matter **(B)** with perinatal characteristic and brain MRI abnormality at TEA.

**A. Gray matter**	**NAA/Cho (mean+/–SD)**	***p–*value**	**NAA/Cr (mean+/–SD)**	***p*–value**	**Cho/Cr (mean+/–SD)**	***p–*value**	**Lac/Cr (mean+/–SD)**	***p*–value**
Antenatal magnesium sulfate	0.60 (0.10)	0.26	1.35 (0.32)	0.71	2.24 (0.50)	0.30	0.00 (0)	0.55
No antenatal magnesium sulfate	0.75 (0.15)		1.30 (0.30)		1.78 (0.51)		0.12 (0.19)	
ACS, yes	0.73 (0.15)	0.23	1.32 (0.31)	0.45	1.79 (0.53)	0.89	0.11 (0.19)	0.35
ACS, no	0.69 (0.11)		1.19 (0.14)		1.78 (0.35)		0.13 (0.13)	
24–26 Weeks GA	0.71 (0.10)	0.54	1.32 (0.31)	0.45	1.89 (0.52)	0.24	0.17 (0.25)	0.63
27–32 Weeks GA	0.74 (0.17)		1.27 (0.31)		1.75 (0.48)		0.09 (0.16)	
Weight birth ≤1,000 g	0.73 (0.12)	0.72	1.34 (0.28)	0.09	1.87 (0.47)	0.08	0.11 (0.19)	0.88
Weight birth >1,000 g	0.74 (0.18)		1.24 (0.32)		1.71 (0.52)		0.11 (0.18)	
**MRI data**								
^1^WM score ≤5	0.72 (0.16)	0.83	1.33 (0.32)	0.28	1.88 (0.4)	0.14	0.12 (0.18)	0.52
WM score >5	0.74 (0.15)		1.28 (0.21)		1.76 (0.4)		0.11 (0.17)	
^2^GM score ≤3	0.75 (0.15)	0.25	1.29 (0.33)	0.84	1.71 (0.30)	0.74	0.09 (0.15)	0.52
GM score >3	0.73 (0.16)		1.29 (0.29)		1.83 (0.57)		0.12 (0.19)	
DESHI	0.71 (0.14)	0.33	1.25 (0.32)	0.17	1.81 (0.58)	0.65	0.10 (0.16)	0.76
No DESHI	0.78 (0.18)		1.35 (0.27)		1.74 (0.29)		0.13 (0.21)	
^3^Lateral ventricular dilation	0.73 (0.17)	0.48	1.27 (0.30)	0.82	1.78 (0.51)	0.96	0.11(0.17)	0.79
No Lateral ventricular dilation	0.75 (0.09)		1.29 (0.29)		1.75 (0.47)		0.11 (0.21)	
^4^Normal TCD	0.74 (0.16)	0.29	1.29 (0.30)	0.97	1.74 (0.5)	0.23	0.10 (0.18)	0.26
Reduction TCD	0.69 (0.14)		1.24 (0.27)		1.82 (0.5)		0.13 (0.16)	
^5^Normal BPD	0.76 (0.18)	0.90	1.33 (0.31)	0.31	1.81 (0.54)	0.78	0.07 (0.17)	0.05
Reduction BPD	0.72 (0.13)		1.23 (0.27)		1.74 (0.46)		0.15 (0.19)	
^6^Normal IHD	0.69 (0.14)	0.16	1.27 (0.21)	0.83	1.95 (0.64)	0.19	0.15 (0.17)	0.1
Increased IHD	0.76 (0.16)		1.28 (0.33)		1.69 (0.36)		0.09 (0.19)	
^7^Normal deep GM volume	0.74 (0.16)	0.75	1.27 (0.29)	0.94	1.76 (0.45)	0.68	0.10(0.16)	0.24
Reduction deep GM volume	0.71 (0.26)		1.3 (0.32)		2.04 (1.21)		0.34 (0.38)	
**Cerebral maturation score, (OR)	0.04	0.02	0.047	0.72	0.02	0.89	0.03	0.8
**B. White matter**								
Antenatal magnesium sulfate	0.70 (0.13)	0.75	1.86 (0.70)	0.36	2.62 (0.82)	0.42	0.00	0.53
No antenatal magnesium sulfate	0.68 (0.14)		1.57 (0.68)		2.3 (0.90)		0.20 (0.29)	
ACS, yes	0.69 (0.13)	0.83	1.61 (0.71)	0.75	2.33 (0.90)	0.95	0.16 (0.23)	0.07
ACS, no	0.68 (0.14)		1.67 (0.86)		2.45 (1.19)		0.40 (0.44)	
24–26 weeks GA	0.72 (0.15)	0.83	1.70 (0.85)	0.61	2.34 (1.02)	0.98	0.17 (0.27)	0.75
27–32 Weeks GA	0.68 (0.13)		1.56 (0.67)		2.31 (0.91)		0.19 (0.28)	
Weight birth ≤1,000 g	0.68 (0.10)	0.59	1.49 (0.57)	0.44	2.19 (0.88)	0.56	0.16 (0.24)	0.69
Weight birth >1,000 g	0.70 (0.16)		1.69 (0.80)		2.41 (0.99)		0.21 (0.30)	
*MRI								
^1^WM score ≤5	0.70 (0.16)	0.90	1.78 (0.86)	0.57	2.53 (1.14)	0.27	0.24 (0.43)	0.81
WM score >5	0.69 (0.13)		1.54 (0.64)		2.25 (0.89)		0.18 (0.24)	
^2^GM score ≤3	0.71 (0.14)	0.25	1.79 (0.93)	0.27	2.52 (1.21)	0.47	0.19 (0.35)	0.42
GM score >3	0.68 (0.13)		1.48 (0.52)		2.20 (0.77)		0.19 (0.24)	
DESHI	0.65 (0.12)	0.02	1.59 (0.72)	0.89	2.39 (0.90)	0.05	0.19 (0.30)	0.88
No DESHI	0.75 (0.14)		1.55 (0.60)		2.12 (0.98)		0.19 (0.24)	
^3^Lateral ventricular dilation	0.70 (0.12)	0.05	1.62 (0.72)	0.14	2.34 (1.00)	0.58	0.20 (0.30)	0.93
No lateral ventricular dilation	0.63 (0.14)		1.31 (0.44)		2.07 (0.57)		0.19 (0.24)	
^4^Normal TCD	0.70 (0.13)	0.21	1.64 (0.76)	0.32	2.35 (1.0)	0.91	0.22 (0.31)	0.69
Reduction TCD	0.65 (0.11)		1.38 (0.47)		2.14 (0.68)		0.16 (0.23)	
^5^Normal BPD	0.70 (0.14)	0.92	1.61 (0.74)	1	2.32 (1.00)	0.91	0.14 (0.22)	0.11
Reduction BPD	0.67 (0.12)		1.51 (0.63)		2.24 (0.87)		0.26 (0.33)	
^6^Normal IHD	0.71 (0.11)	0.14	1.64 (0.82)	0.73	2.29 (1)	0.91	0.23 (0.38)	0.95
Increased IHD	0.67 (0.13)		1.51 (0.61)		2.27 (0.91)		0.18 (0.23)	
^7^Normal deep GM volume	0.788 (0.12)	0.15	1.25 (0.30)	0.54	1.66 (0.62)	0.36	0.00 (0.00)	0.14
Reduction deep GM volume	0.68 (0.13)		1.70 (0.70)		2.31 (0.94)		0.21 (0.29)	
** Cerebral maturation score (OR)	0.4	0.02	0.12	0.34	−0.22	0.098	−0.18	0.88

ACS, antenatal corticosteroid; GA, gestational age, GM, gray matter; WM, white matter; DEHSI, diffuse excessive high signal intensity; TCD, Trans Cerebellum Diameter; BPD, biparietal diameter; IHD, interhemispheric diameter; DGM, deep gray matter; NAA, N Acetyl Aspartate; Cr, creatine; Cho, choline; Lac, lactate.

^**^Correlations between MRS ratios and cerebral maturation score (Total maturation score, sum of: Myelination Score (1–7), Cortical infolding Score (1–6), Germinal Matrix Score (1–4), Bands of migrating glial cells Score (l1–l4). The higher the score, the more advanced the maturation).

^*^MRI Data:

Total maturation score, sum of Myelination Score (1–7), Cortical infolding Score (1–6), Germinal Matrix Score (1–4), Bands of migrating glial cells Score (l1–l4). The higher the score, the more advanced the maturation.

Brain abnormality was determined using a rating system described previously (16). The scoring system gives an overall rating of white matter, cortical gray matter, deep gray matter, and cerebellar abnormality.

^1^The WM score, abnormal was between 6–17; ^2^The CGM score, abnormal if between 3–9. Measures, patterns of impaired brain growth moderate to severe were identified; ^3^ventricular dilations, bilateral or unilateral ≥ 10mm; ^4^TCD, Corrected trans cerebellar diameter, ≤ 47mm; ^5^BPD /WM Vol, Biparietal corrected diameter, <72. 5; ^6^(IHD Extra cerebral space), Interhemispheric distance (IHD)≥ 5 mm; ^7^Deep GM volume, diameter gray matter, ^8^ The cortical gray matter scale (CGM) was the sum of three subscales with a score of 0 to 3 (Inder, Wells, Mogridge, Sencer and Volpe 2003), (1) Signal abnormality, (2) Delayed gyral maturation, and (3) Increased extra cerebral space.

The CGM scale was normal if ≤3, slightly abnormal if the score was between 4–6, and moderately to severely abnormal if between 7–9. We have grouped them into two categories, normal ≤3 and abnormal >or equal to 3. The cerebral white matter scale (CWM) is the sum of six subscales,

(1) Presence and severity of cystic lesions, (2) Signal abnormality, (3) Thinning of corpus callosum, (4) Lateral ventricular dilation, (5) Periventricular white matter loss, and (6) Punctate lesions. Each item was rated from 0 to 3, except for punctate lesions which were rated from 0 to 2. The CWM score was considered normal if ≤5, slightly abnormal if it was between 6–11, and moderately to severely abnormal if it was between 12–17 (4). We have grouped them into two categories, normal≤5 and abnormal > to 5.

Comparisons of means of metabolite ratios between different categories of qualitative variables by Mann Whitney.

P<0.05. Variables are expressed as means and standard deviations; numbers in brackets are ranges. Bold indicates a significant P–value.

### 1H-MRS metabolites and neurodevelopment in preterm infants at 24 months

There was a significant correlation, independent of MRI brain lesions at TEA, in the multivariate analysis in the white matter between the reduction in the NAA/Cho ratio and abnormal communication [OR: 0.003[0.000–0.95], *p* = 0.04], and only the trend between the increase in the Lac/Cr ratio and a fine motor anomaly [OR: 39.35 [0.003–33.57, *p* = 0.03], and between the reduction in the NAA/Cho ratio and abnormal ASQ [OR: 0.005 [0.000–1.19], *p* = 0.05] (Table 6).

**Table 6:** Correlations between NAA/Cho, NAA/Cr, and Cho/Cr / Lac/Cr ratio (GM and WM) with ASQ, in univariate analysis.

**Gray matter**	**NAA/Cho**			**NAA/Cr**			**Cho/Cr**			**Lac/Cr**		
	***N***	***P***	**OR IC (95%)**	***N***	***P***	**OR IC (95%)**	***N***	***P***	**OR IC (95%)**	***N***	***P***	**OR IC (95%)**
Communication	51	0.28	0.11 [0.002–6.12]	51	0.50	0.54 [0.090–3.26]	51	0.99	1.00 [0.341–2.96]	51	0.90	1.25 [0.03–50.59]
Gross motor	51	0.36	0.04 [0.000–33.35]	51	0.30	0.16 [0.006–4.96]	51	0.63	0.61 [0.080–4.699]	51	0.59	4.00 [.025–644.59]
Fine motor	50	0.75	0.38 [0.001–166.64]	50	0.07	0.02 [0.000–1.45]	51	0.12	0.08 [0.004–1.927]	51	0.91	1.37 [0.004–471.83]
Problem solving	51	0.99	0.97 [0.012–79.95]	53	0.12	0.11 [.007–1.82]	51	0.14	0.20 [0.026–1.675]	51	0.45	4.90 [0.07– 310.91]
Personal and social skills	51	0.18	0.02 [0.000–5.51]	51	0.59	0.52 [0.05–5.54]	51	0.88	1.10 [.285–4.29]	51	1.94	1.94 [0.023 164.92]
ASQ total		0.68	0.780 [0.231–2.629]		0.76	1.061 [0.720–1.562]		0.92	1.010 [0.825–1.236]			
**White matter**												
Communication	55	0.06	0.01 [0.000–1.254]	55	0.38	0.69 [0.303–1.59]	55	0.85	0.94 [0.52–1.71]	55	0.65	1.55 [0.22–10.65]
Gross motor	54	0.64	0.20 [0.000–185.768]	54	0.64	0.20 [0.000–185.76]	54	0.43	0.60 [0.16–2.16)	54	0.49	0.181[0.001–23.00]
Fine motor	55	0.66	0.22.[0.000–213.902]	55	0.66	0.22 [0.000–213.90]	55	0.73	1.16 [0.47–2.82]	55	0.03	14.98 [1.158–193.87]
Problem solving	55	0.81	047[0.001–231.977]	55	0.81	0.47 [0.001–231.97]	55	0.33	0.53 [0.14–1.91]	55	0.77	1.48 [0.09–22.90]
Personal and social skills	55	0.94	1.19 [0.007–198.078]	55	0.94	1.19 [0.007–198.07]		0.55	1.23 [0.63–2.45]	55	0.46	2.29 (0.25–20.93]
ASQ total		0.06	0.489 [0.231–1.037]		0.96	0.487 [0.854–1.080]	55	0.87	1.007 [0.925–1.096]			

P, p–value; NAA, N Acetyl Aspartate; Cr, Creatine; Cho, Choline; Lac, Lactate.

OR, represents the correlation between the MRS (magnetic resonance spectroscopy) ratios and the pathological ASQ score.

ASQ, age and stage questionnaire; DEHSI, diffuse excessive high signal intensity.

Variables are expressed as means ± standard deviations; numbers in brackets are ranges.

In the gray matter (Table 7), two borderline significant results were noted and independent of the MRI brain lesions at TEA (1) between the reduction in the NAA/Cho ratio and an anomaly in sociability [0.006 [0.000–2.74], *p* = 0.1], and (2) between the increase in the Lac/Cr ratio [90.72 [0.0365–22,570.95], *p* = 0.1] and a problem-solving anomaly.

**Table 7:** Correlations between NAA/Cho. NAA/Cr and Cho/Cr / Lac/Cr ratio (GM and WM) with ASQ, in multivariate analysis.

**Gray Matter**	**NAA/Cho**			**NAA/Cr**			**Cho/Cr**			**Lac/Cr**		
	***N***	***P***	**OR IC (95%)**	***N***	***P***	**OR IC (95%)**	***N***	***P***	**OR IC (95%)**	***N***	***P***	**OR IC (95%)**
Communication	48	0.19	0.058 [0.001–4.38]	48	0.33	0.37 [0.052–2.75]	48	0.95	1.035[.336–3.18]	48	0.43	5.04 [0.08–288.46]
Gross motor	48	0.35	0.028 [0.000–52.02]	48	0.48	0.25 [0.005–12.38]	48	0.91	0.89 [.118–6.822]	48	0.35	15.14 [0.048 −4,817.57]
Fine motor												
Problem solving	48	0.95	0.87 [0.008–93.81]	48	0.23	0.17 [.010–3.055]	48	0.26	0.31 [0.039–2.46]	48	0.10	90.72 [0.0365 −22,570.95]
Personal and social skills	48	0.10	0.006 [0.000–2.74]	48	0.28	0.18 [0.008–4.07]	48	0.97	1.02 [.225–4.70]	48	0.54	4.23 [0.04– 450.04]
ASQ total	47	0.64	0.042[0.001– 35,112.27]	50	0.26	0.36.[0.061–2.4]	48	0.408	0.61 [0.18 −1.94]	50	0.40	0.61 [0.18–1.94]
**White matter**												
Communication	51	0.04	0.003[0.000–0.95]	48	0.43	0.67 [0.248–1.828]	48	0.77	0.90 [0.468– 1.76]	48	0.512	1.97.[0.26– 14.92]
Gross motor	51	0.70	0.18 [0.000–1,031.22]	48	0.70	0.18 [0.000 1,031.22]	48	0.63	0.72 [0.19– 2.68]	48	0.61	0.29.[0.26 14.92]
Fine motor	51	0.76	0.27 [0.00–1,390.69]		0.85	1.11 [0.343– 3.64]		0.65	1.23 [0.487– 3.141]		0.02	39.35 [0.003 33.57]
Problem solving	51	0.72	4.31 [0.001–14,163.8]	48	0.43	0.48 [0.07– 3.03]	48	0.72	4.31 [0.001 14,163.87]	48	0.51	2.53 [1.777– 871.30]
Personal and social skills	51	0.61	5.29 [0.008–3,660.75]	48	0.51	1.40 [0.50– 3.93]	48	0.61	5.29 [0.008– 3,660.75]	48	0.25	3.79 [3.7 37.22]
ASQ total	50	0.049	0.005 [0.000– 1.19]	50	0.23	0.575[0.23– 1.42]	50	0.44	0.78 [0.41 1.46]	50	0.36	2.567[0.03 19.78]

NAA, N Acetyl Aspartate; Cr, Creatine; Cho, Choline; Lac, Lactate.

Multivariate analysis, a forced adjustment was performed.

GA and variables related to at least one of the ratios (in Tables 5A,B at threshold p <0.10) (PN and WM volume reduction for GM) and DESHI and lateral ventricular dilation for WM.

OR, represents the correlation between the MRS (magnetic resonance spectroscopy) ratios and the pathological ASQ score.

## Discussion

In this study, 1H-MRS spectroscopy performed at TEA is predictive of neurodevelopment at 24 months, independent of MRI abnormalities.

In the posterior periventricular white matter, there is a correlation between (1) the reduction in the NAA/Cho ratio and abnormal neurodevelopment (ASQ) and/or communication, and (2) an increase in the Lac/Cr ratio and fine motor anomaly.

In the gray nuclei, there is a trend between (1) the reduction in the NAA/Cho ratio and sociability disorders, and (2) the increase in the Lac/Cr ratio and an anomaly in problem-solving.

Although a recent review confirmed the predictive value of 1H-MRS for the assessment of neurodevelopment in premature infants, its use in clinical practice is not common (27). There are, however, only 20 studies selected, and few subjects, along with several limitations such as (1) 1H-MRS techniques varied including different echo time (TEs), magnetic field strengths, preferred regions, and voxel size, which may have affected tissue particularity and resulted in regions of interest that included non-targeted tissues; (2) the population analyzed was heterogeneous, and (3) different endpoints of neurobehavioral outcomes were assessed.

The methods of acquisition, post-processing, and analysis have an important impact on the interpretation of the results. Recent recommendations on the treatment of single-voxel 1H-MRS data, as used in our study, have been established by an expert working group to increase quality and enable reproducibility (28).

In our study, the field intensity was carried out using a 1.5 T system, or even, for some newer machines, 3.0 T equipment which can improve the identification and quantification of metabolites. In general, for major signals in the brain such as NAA, Cho, and Lac, the use of 1.5 T or 3.0 T does not change the interpretation of the results (28). Similarly, the practice of a long echo, as in our study, discriminates lactate from lipids and overlapping multiplets in the spectrum. This technique makes it possible to obtain good quality NAA/Cho and NAA/Cr ratios, and therefore obtain good reproducibility and comparability (29).

Finally, the comparison of ratios of metabolite levels, and not the absolute metabolite concentrations, makes it possible to eliminate the effect of a difference in treatment methods on clinically relevant parameters. Our volume of interest (VOI) is compliant in order to have tissue specificity. In our study, the posterior periventricular white matter and the basal ganglia and thalamus are the chosen areas of interest because their metabolism is frequently altered at the TEA in premature babies. Even in our study, we found little association between the metabolic ratios in both white matter and gray matter and MRI lesions. We did find that (1) the increase in the NAA/Cho ratio in gray and white matter was inversely related to the score of maturation, (2) the increase in the Lac/Cr ratio in the GM was linked to abnormalities in the volume of the white matter, and (3) there was a link between the decrease of the NAA/Cho ratio in the WM and the presence of a white matter hyper signal (DESHI), which illustrates the intricacy between metabolism, maturation, and differentiation, and confirms the relevance of our areas of interest and the selected metabolites.

However, this study has several limitations. The sample size was relatively small and nested in a population-based study. Given the exploratory nature of the study, a prospective study with a larger sample size is required to validate the results of our study. The MRI was performed at different periods (37 to 42 weeks). There is no comparison group with term infants. Normal development at 24 months of corrected age may not reflect normal motor, cognitive, and language functions at older ages. Generally, higher-order or more subtle brain dysfunction will not be evident until later on in childhood.

In the literature, we found only four studies using the 1H-MRS at TEA in association with the neurodevelopmental outcomes of preterm infants at 24 months like our study (14). Within this small sample, only 130 children had been assessed at 24 months, two studies showed no association with neurodevelopment, contrary to our study (30, 31). The heterogeneity of studies may explain the difference in the results: the size of the Voxels between 1 cm^2^ and 4 cm^2^), a single study with a long echo like our study (31), regions of interest explored different gray matter only (31), supraventricular area and cortex (23), periventricular zone and gray nuclei area (30), cerebellum (32). The heterogeneity of assessments at 24 months; the Bayley score (23, 31, 32), and the Griffiths score (30), can make comparisons difficult. However, a metabolic study of frontal white matter and cingulate gyrus cortex in a population of 177 preterm newborns with 1H-MRS, at two time points of 32 and 40 weeks TEA GA, showed a slower increase in NAA/Cho (or decrease), similar to our study, and correlates with motor and cognitive impairment at 18 months (33). Recently, Hyodo et al. found reduced NAA/Cho ratios in the frontal white matter of preterm infants with normal MRIs as compared to term infants along with a significantly lower NAA/Cho ratio in the thalamus correlated with mild developmental delay at 18 months (34). In our study, there is a trend between the decrease in the NAA/Cho ratio in GM and sociability disorders. Finally, the NAA/Cho ratio in premature babies at TEA, regardless of the area of interest, the ratio is most often correlated with long-term outcomes (14). A meta-analysis showed that the Lac/NAA ratio in the deep gray matter was an accurate biomarker for predicting abnormal neurodevelopmental outcomes in the hypoxic-ischemic encephalopathy (35). Similarly, in our work, there is a significantly close link between the Lac/Cr ratio in the gray matter and problem-solving. In one study an elevated Lac peak in the white matter in the preterm infant was significantly associated with fine motor scores, (36). However, in our study, there was an increase in the Lac /Cr ratio.

## Conclusion

This study confirms that the use of NAA in preterm infants as a biomarker of neurodevelopment has great potential given the almost exclusive presence of NAA in immature neurons and proligodendrocytes-cells that are particularly vulnerable. Multicenter population-based studies in preterm infants are needed to confirm the results with monovoxel metabolic profiles according to recent recommendations (29) in order to obtain detailed information on metabolites in the posterior periventricular zone, the deep gray matter, and the cerebellum, known to be areas at risk in preterm newborns at TEA. Similarly, the presence of lactates is abnormal in preterm infants at TEA.

## Data availability statement

The raw data supporting the conclusions of this article will be made available by the authors, without undue reservation.

## Ethics statement

The studies involving human participants were reviewed and approved by PHRC EPIRMEX. Written informed consent to participate in this study was provided by the participants' legal guardian/next of kin.

## EPIRMEX study group

Catherine Adamsbaum, Pierre-Yves Ancel, Catherine Arnaud, Olivier Baud, Nathalie Bednarek, Valérie Biran, Olivier Brissaud, Aude Charollais, Katia Chaumoitre, Thierry Debillon, Denis Devictor, Patrice Gillet, Catherine Gire, Bernard Guillois, Sophie Guyetant, Jean-Michel Hascoet, Petra Huppi, Stéphane Marret, Sylvie Nguyen, Véronique Pierrat, Patrick Pladys, Jean Christophe Roze, Umberto Simeoni, Dominique Sirinelli, and Véronique Zupan.

## Author contributions

Guarantors of the integrity of the entire study and literature research: CG, BT, JB, and SM. Study concepts/study design or data acquisition or data analysis/interpretation, manuscript drafting or manuscript revision for important intellectual content, agrees to ensure any questions related to the work are appropriately resolved, and manuscript editing: all authors. Clinical studies: CG and BT. Statistical analysis: JB and CG. All authors contributed to the article and approved the submitted version.

## Funding

This work has been supported by PHRC EPIRMEX, ancillary cohort EPIPAGE 2. This work also supported by institutional grants from the French 2014 Appel d'Offre Recherche Clinique Assistance Publique, H4pitaux de Marseille (number UD/IM/55/2015).

## Conflict of interest

The authors declare that the research was conducted in the absence of any commercial or financial relationships that could be construed as a potential conflict of interest.

## Publisher's note

All claims expressed in this article are solely those of the authors and do not necessarily represent those of their affiliated organizations, or those of the publisher, the editors and the reviewers. Any product that may be evaluated in this article, or claim that may be made by its manufacturer, is not guaranteed or endorsed by the publisher.

## References

[1] Panigrahy A Wisnowski JL Furtado A Lepore N Paquette L Bluml S. Neuroimaging biomarkers of preterm brain injury: toward developing the preterm connectome. Pediatr Radiol Jan. (2012) 42 (Suppl 1):S33–61. 10.1007/s00247-011-2239-4 22395719 PMC4517479

[2] Pierrat V Marchand-Martin L Arnaud C Kaminski M Resche-Rigon M Lebeaux C. Neurodevelopmental outcome at 2 years for preterm children born at 22 to 34 weeks' gestation in France in 2011: EPIPAGE-2 cohort study. BMJ. (2017) 358:j3448. 10.1136/bmj.j3448 28814566 PMC5558213

[3] Ni Y Mendonça M Baumann N Eves R Kajantie E Hovi P. Social functioning in adults born very preterm: individual participant meta-analysis. Pediatrics Nov. (2021) 148:e2021051986. 10.1542/peds.2021-051986 34702720

[4] Woodward LJ Clark CAC Bora S Inder TE. Neonatal white matter abnormalities an important predictor of neurocognitive outcome for very preterm children. PLoS ONE. (2012) 7:51879. 10.1371/journal.pone.0051879 23284800 PMC3532310

[5] Miller SP Ferriero DM Leonard C Piecuch R Glidden DV Partridge JC. Early brain injury in premature newborns detected with magnetic resonance imaging is associated with adverse early neurodevelopmental outcome. J Pediatr. (2005) 147:609–16. 10.1016/j.jpeds.2005.06.033 16291350

[6] Garbi A Sorin G Coze S Resseguier N Brévaut-Malaty V Marret S. Predictive value of brain MRI at term-equivalent age in extremely preterm children on neurodevelopmental outcome at school-age. Brain Imaging Behav. (2022) 16:878–87. 10.1007/s11682-021-00559-9 34661873

[7] Kimura H Fujii Y Itoh S Matsuda T Iwasaki T Maeda M. Metabolic alterations in the neonate and infant brain during development: evaluation with proton MR spectroscopy. Radiology. (1995) 194:483–9. 10.1148/radiology.194.2.7529934 7529934

[8] Girard N. Magnetic resonance spectroscopy for cerebral imaging. Arch Pediatr. (2010) 17:731–2. 20654864 10.1016/S0929-693X(10)70082-8

[9] Alkan A Kutlu R Sigirci A Baysal T Altinok T Yakinci C. Giant axonal neuropathy: MRS findings. J Neuroimaging. (2003) 13:371–5. 10.1111/j.1552-6569.2003.tb00208.x 14569833

[10] Wisnowski JL Schmithorst VJ Rosser T Paquette L Nelson MD Haynes RL. Magnetic resonance spectroscopy markers of axons and astrogliosis in relation to specific features of white matter injury in preterm infants. Neuroradiology. (2014) 56:771–9. 10.1007/s00234-014-1380-9 24903580 PMC9242581

[11] Koob M Viola A Le Fur Y Viout P Ratiney H Confort-Gouny S. Creatine, glutamine plus glutamate, and macromolecules are decreased in the central white matter of premature neonates around term. PLoS ONE. (2016) 11:e0160990. 10.1371/journal.pone.0160990 27547969 PMC4993494

[12] Brossard-Racine M Murnick J Bouyssi-Kobar M Coulombe J Chang T Limperopoulos C. Altered cerebellar biochemical profiles in infants born prematurely. Sci Rep. (2017) 7:8143. 10.1038/s41598-017-08195-4 28811513 PMC5557848

[13] Gasparovic C Caprihan A Yeo RA Phillips J Lowe JR Campbell R. The long-term effect of erythropoiesis stimulating agents given to preterm infants: a proton magnetic resonance spectroscopy study on neurometabolites in early childhood. Pediatr Radiol. (2018) 48:374–82. 10.1007/s00247-017-4052-1 29335880 PMC5823776

[14] Morel B Bertault P Favrais G Tavernier E Tosello B Bednarek N. Automated brain MRI metrics in the EPIRMEX cohort of preterm newborns: Correlation with the neurodevelopmental outcome at 2 years. Diagn Interv Imaging. (2021) 102:225–32. 10.1016/j.diii.2020.10.009 33187906

[15] Inder TE Wells SJ Mogridge NB Spencer C Volpe JJ. Defining the nature of the cerebral abnormalities in the premature infant: a qualitative magnetic resonance imaging study. J Pediatr. (2003) 143:171–9. 10.1067/S0022-3476(03)00357-3 12970628

[16] Kidokoro H Anderson PJ Doyle LW Neil JJ Inder TE. High signal intensity on T2-weighted MR imaging at term-equivalent age in preterm infants does not predict 2-year neurodevelopmental outcomes. AJNR Am J Neuroradiol. (2011) 32:2005–10. 10.3174/ajnr.A2703 21960493 PMC7964405

[17] Volpe JJ. The encephalopathy of prematurity–brain injury and impaired brain development inextricably intertwined. Semin Pediatr Neurol. (2009) 16:167–78. 10.1016/j.spen.2009.09.005 19945651 PMC2799246

[18] Fleiss B Gressens P Stolp HB. Cortical gray matter injury in encephalopathy of prematurity: link to neurodevelopmental disorders. Front Neurol. (2020) 11:575. 10.3389/fneur.2020.00575 32765390 PMC7381224

[19] Boardman JP Craven C Valappil S Counsell SJ Dyet LE Rueckert D. A common neonatal image phenotype predicts adverse neurodevelopmental outcome in children born preterm. NeuroImage. (2010) 52:409–14. 10.1016/j.neuroimage.2010.04.261 20451627

[20] Ball G Pazderova L Chew A Tusor N Merchant N Arichi T. Thalamocortical connectivity predicts cognition in children born preterm. Cereb Cortex. (2015) 25:4310–8. 10.1093/cercor/bhu331 25596587 PMC4816783

[21] Girard N Chaumoitre K Confort-Gouny S Viola A Levrier O. Magnetic resonance imaging and the detection of fetal brain anomalies, injury, and physiologic adaptations. Curr Opin Obstet Gynecol. (2006) 18:164–76. 10.1097/01.gco.0000193002.58158.07 16601478

[22] Xu D Bonifacio SL Charlton NN P Vaughan C Lu Y Ferriero DM. MR spectroscopy of normative premature newborns. J Magn Reson Imaging. (2011) 33:306–11. 10.1002/jmri.22460 21274971 PMC3391540

[23] Bapat R Narayana PA Zhou Y Parikh NA. Magnetic resonance spectroscopy at term-equivalent age in extremely preterm infants: association with cognitive and language development. Pediatr Neurol. (2014) 51:53–9. 10.1016/j.pediatrneurol.2014.03.011 24938140 PMC5942892

[24] Pouwels PJ Brockmann K Kruse B Wilken B Wick M Hanefeld F. Regional age dependence of human brain metabolites from infancy to adulthood as detected by quantitative localized proton MRS. Pediatr Res. (1999) 46:474–85. 10.1203/00006450-199910000-00019 10509371

[25] de Beer R van den Boogaart A van Ormondt D Pijnappel WW den Hollander JA Marien AJ. Application of time-domain fitting in the quantification of *in vivo* 1H spectroscopic imaging data sets. NMR Biomed. (1992) 5:171–8. 10.1002/nbm.1940050403 1449952

[26] Vanhamme L van den Boogaart A Van Huffel S. Improved method for accurate and efficient quantification of MRS data with use of prior knowledge. J Magn Reson. (1997) 129:35–43. 10.1006/jmre.1997.1244 9405214

[27] Cebeci B Alderliesten T Wijnen JP van der Aa NE Benders MJNL de Vries LS. Brain proton magnetic resonance spectroscopy and neurodevelopment after preterm birth: a systematic review. Pediatr Res. (2022) 91:1322–33. 10.1038/s41390-021-01539-x 33953356

[28] Alderliesten T de Vries LS Staats L van Haastert IC Weeke L Benders MJNL. MRI and spectroscopy in (near) term neonates with perinatal asphyxia and therapeutic hypothermia. Arch Dis Child Fetal Neonatal Ed. (2017) 102:F147–52. 10.1136/archdischild-2016-310514 27553589

[29] Near J Harris AD Juchem C Kreis R Marjańska M Öz G. Preprocessing, analysis and quantification in single-voxel magnetic resonance spectroscopy: experts' consensus recommendations. NMR Biomed May. (2021) 34:e4257. 10.1002/nbm.4257 32084297 PMC7442593

[30] Roelants-van Rijn AM van der Grond J Stigter RH de Vries LS Groenendaal F. Cerebral structure and metabolism and long-term outcome in small-for-gestational-age preterm neonates. Pediatr Res. (2004) 56:285–90. 10.1203/01.PDR.0000132751.09067.3F 15181199

[31] Augustine EM Spielman DM Barnes PD Sutcliffe TL Dermon JD Mirmiran M. Can magnetic resonance spectroscopy predict neurodevelopmental outcome in very low birth weight preterm infants? J Perinatol. (2008) 28:611–8. 10.1038/jp.2008.66 18615089 PMC2844764

[32] Van Kooij BJM Benders MJNL Anbeek P Van Haastert IC De Vries LS Groenendaal F. Cerebellar volume and proton magnetic resonance spectroscopy at term, and neurodevelopment at 2 years of age in preterm infants. Dev Med Child Neurol. (2012) 54:260–6. 10.1111/j.1469-8749.2011.04168.x 22211363

[33] Chau V Synnes A Grunau RE Poskitt KJ Brant R Miller SP. Abnormal brain maturation in preterm neonates associated with adverse developmental outcomes. Neurology. (2013) 81:2082–9. 10.1212/01.wnl.0000437298.43688.b9 24212394 PMC3863348

[34] Hyodo R Sato Y Ito M Sugiyama Y Ogawa C Kawai H. Magnetic resonance spectroscopy in preterm infants: association with neurodevelopmental outcomes. Arch Dis Child Fetal Neonatal Ed. (2018) 103:F238–44. 10.1136/archdischild-2016-311403 28724545

[35] Thayyil S Chandrasekaran M Taylor A Bainbridge A Cady EB Chong WKK. Cerebral magnetic resonance biomarkers in neonatal encephalopathy: a meta-analysis. Pediatrics Febr. (2010) 125:e382–395. 10.1542/peds.2009-1046 20083516

[36] Hart AR Smith MF Whitby EH Alladi S Wilkinson S Paley MN. Diffusion-weighted imaging and magnetic resonance proton spectroscopy following preterm birth. Clin Radiol. (2014) 69:870–9. 10.1016/j.crad.2014.04.001 24935906

