# Supplementary material for: A correlation between Magnetic Resonance Spectroscopy (1-H MRS) and the neurodevelopment of two-year-olds born preterm in an EPIRMEX cohort study
Source: Front Pediatr. 2022 Aug 19;10:936130. doi: 10.3389/fped.2022.936130 (PMC9437452; doi:10.3389/fped.2022.936130)
Supplement: Supplementary file 1 [file Data_Sheet_1.PDF]

Appendix1

Standardized Scoring System

BRAIN MATURATION

Myelination

1: M1 Myelination evident in brain stem, cerebellar peduncle, inferior colliculus, cerebellar vermis;
2 : M2 = M1 + subthalamic nuclei, globus pallidus, ventrolateral thalamus;
3: M3 = M2 + caudal portion of the posterior limb of the internal capsule (PLIC);
4: M4 = M3 + complete PLIC;
5: M5 = M4 + optic radiation;
6 : M6 = M5 + corona radiata;
7 : M7 = M6 + anterior limb of internal capsule

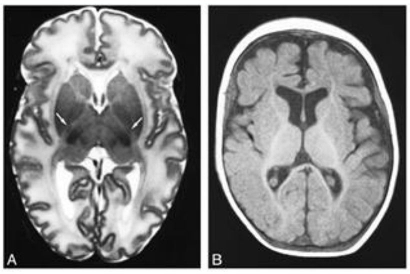

Fig 2. Myelination.
A, Axial T2-weighted image from an infant of 35 weeks' PMA (GA = 35). Myelination can be seen to have started in the posterior limb of the internal capsule (arrows). Score: GM2, B1, M3, C3; TMS = 9.
B, Axial T1-weighted image from an infant of 52 weeks' PMA, too old for this study and only included to illustrate features of myelination. Myelination is seen in anterior and posterior limbs of internal capsule, together with the optic radiation and corona radiata. The unmyelinated white matter is isointense with the gray matter. Score: GM4, B4, M7, C6; TMS = 21.

Cortical infolding

1: C1 Frontal and occipital cortex completly smooth, insula wide open; thin bright cortical rim on T1, generally low-intensity white matter (WM) on T1
2 : C2 = Frontal cortex still very smooth, some sulci evident in occipital cortex; insula still wide with almost smooth internal surface; WM low intensity on T1;
3 : C3 = Frontal and occipital cortex similar number of convolutions; frontal sulci stillquite shallow; internal surface of insula more convoluted; WM still somewhat low intensity on T1;
4 : C4 = Frontal & occipital cortex folded and rich in sulci; frontal sulci obvious along interhemispheric fissure; occipital WM separated into strands by deeper sulci; insula more convoluted and ...;
5 = C5 Front. & occ. WM separated into stnds by deeper sulci; insula completely infolded; WM still distinguishable from gray matter en T1;
6 : C6 = As above but WM now isointense with gray matter on T1

Fig 4. Cortical infolding.
A, Axial T1-weighted image from an infant of 34 weeks' PMA (GA = 34). The frontal and occipital cortices have a similar number of shallow convolutions, and the internal surface of the insula is developing convolutions. The unmyelinated white matter is of low intensity in relation to gray matter. Score: GM2, B1, M3, C3; TMS = 9.
B, Axial T2-weighted image from an infant of 36 weeks' PMA (GA = 36). The frontal and occipital cortices are folded and quite rich in sulci, with deeper sulci evident along the interhemispheric fissure. The insula is more convoluted, and the posterior white matter shows some separation into strands by deeper sulci than in the frontal cortex. Score: GM2, B2, M3, C4; TMS = 11.

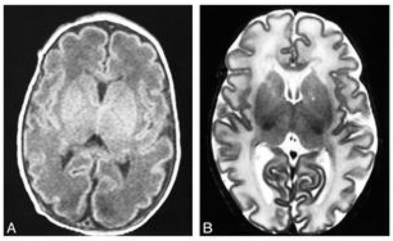

Germinal matrix

4 : G1 = No matrix evident;

3 : G2 = Matrix at anterior horns alone;

2 : G3 = Matrix evident at CTN & anterior horns only;

1 : G4 = Matrix seen in posterior horn, at CTN and anterior

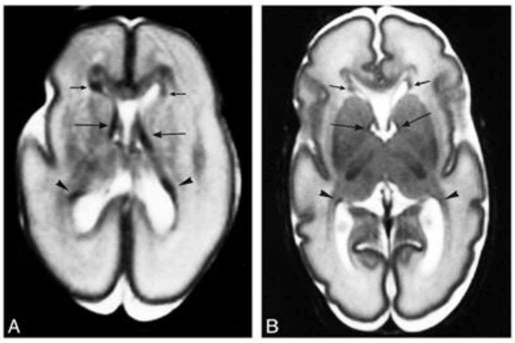

Fig 1. Germinal matrix distribution.
A, Axial T2-weighted image from an infant of 23 weeks' gestation (PMA = 24 weeks). Germinal matrix can be seen in the posterior horns (*arrowheads*), anterior horns (*short arrows*), and at the caudothalamic notch (*long arrows*). Score: GM1, B1, C1, M1; TMS = 4.
B, Axial T2-weighted image from infant of 27 weeks' PMA (GA = 25 weeks). Germinal matrix can be seen in same distribution as in *A*, but is much less evident. Score: GM1, B1, M2, C2; TMS = 6.

Bands of migrating glial cells

4: GI1= No bands seen;

3 : GI2 = Narrow band alone;

2 : GI3 = Broad band alone;

1 : GI4 = broad bands with additional narrower bands

Fig 3. Bands of migrating glial cells.
A, Axial T2-weighted image through foramen of Monro in an infant of 35 weeks' PMA (GA = 35 weeks). A broad band of low signal intensity (*arrows*) with additional narrower bands (*arrowheads*) can be seen in the frontal periventricular white matter. Score: GM2, B1, M2, C2; TMS = 7.
B, Axial T2-weighted image from an infant of 42 weeks' PMA (GA = 42). There is no band of low signal intensity in the frontal periventricular white matter. Score: GM4, B4, M4, C5; TMS = 17.

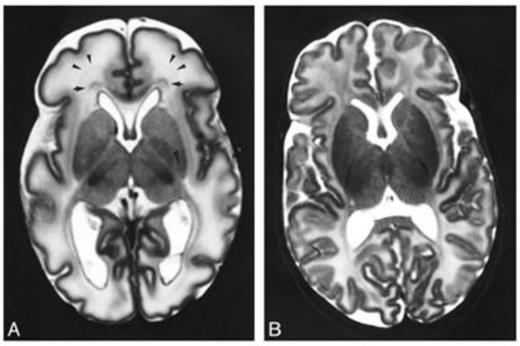

WHITE MATTER INJURY SCORE (Woodward et al, NEJM, 2006)

White matter signal abnormality : T1 and T2 weighted signal abnormalities in the WM best observed in the axial imaging :

1 = normal;

2 = mild abnormality;

3 = moderate-severe abnormality

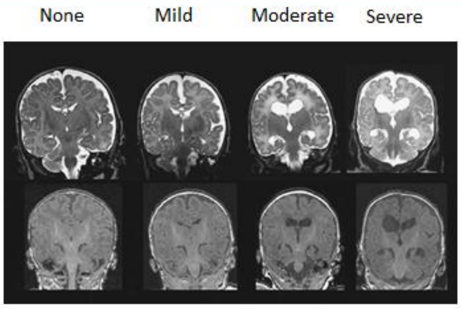

Ventricular dilatation : based on combinend T1-T2 weighted imaging in both axial and sagittal imaging:

1 = normal;
2 = mild dilatation resulting in mild rounding of the frontal…;
3 = moderate-severe enlargement of the frontal, temporal…

Periventricular WM loos : T1 and T2 weighted analysis of the ratio of periventricular WM to ventricular volume in both axial and sagittal imaging :
1 = normal;
2 = mild abnormality;
= moderate-severe abnormality

Cystic abnormalities :
1 = no cysts;
2 = less than 2mm single focal cyst;
3 = multiple cysts or single larger cyst > 2mm

Thinning of corpus callosum: based on the T1 and T2 wieghted imaging:
1 = normal with thick corpus callosum visible in all views;
2 = focal thinning in the corpus callosum often visible in the mid…;
3 = global thinning across the entire corpus callosum

Punctate white matter lesions (PWML)
0 = not present;
1 = present, <6;
2 = present, >=6

GRAY MATTER INJURY SCORE

Presence of gray cortical signal abnormality: high signal intensity in the cortex on axial T1 and/or loss of cortical ribbon signa lon axial T2-weighted MRI
1 = normal;
2 = mild abnormality;
3 = moderate-severe abnormality

Quality of gyral maturation
1 = normal for 40 weeks;
2 = 2 to 4 weeks delay in gyral development (w36-40);
3 = more than 4 weeks delay in gyral development

Size of subarachnoidal space : Score based on the sagittal TA and coronal T1 or T2-weighted MR images for the size of the subarachnoid space in relation to the cerebral mantle
1 = Small subarachnoid space barely visible;
2 = Middly enlarged CSF space with visible enlargement of the…;
3 = More sustantially enlarged global subarachnoid space with…

Lesions involving the cerebellum

1 = normal;
2 = single unilateral left;
3 = single unilateral right;
4 = bilateral;
5 = multiple involving both hemispheres;
6 = associated with volume loss

Cerebellar measurements:
What is the height of cerebellum in mid sagittal view
What is the width of cerebellum in mid sagittal view at level of the 4th ventricle
What is the width of cerebellum in coronal view

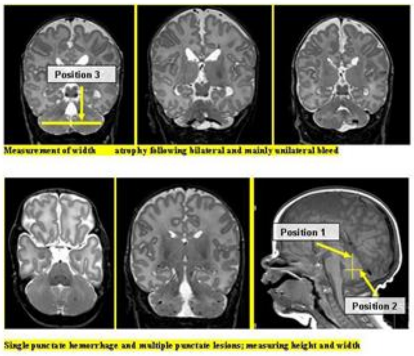

PRESENCE OF DESHI ON T2 WEIGHTED MR IMAGING

0 = No DESHI throughout the white matter;
1 = visible only within the crossroads;
2 = visible in other regions of WM (1 regions);
3 = visible in other regions of WM (2 regions);
4 = visible in other regions of WM (≥3 regions)

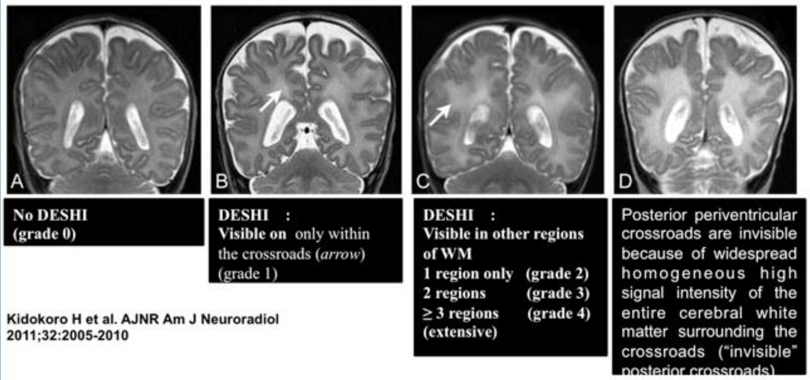

ANATOMICAL READING

Cerebral WM Abnormality

Cystic degeneration

0 = None;
1 = Focal unilateral;
2 = Focal bilateral;
3 = Extensive unilateral;
4 = Extensive bilateral

Help: Representative MR images of regional injury. Axial T1- or T2-weighted MR images demonstrating classification of cystic WM lesions (A–D). Cystic WM lesions are defined by their extent: focal unilateral (arrow, A), focal bilateral (arrows, B), extensive unilateral (C), and extensive bilateral (D).

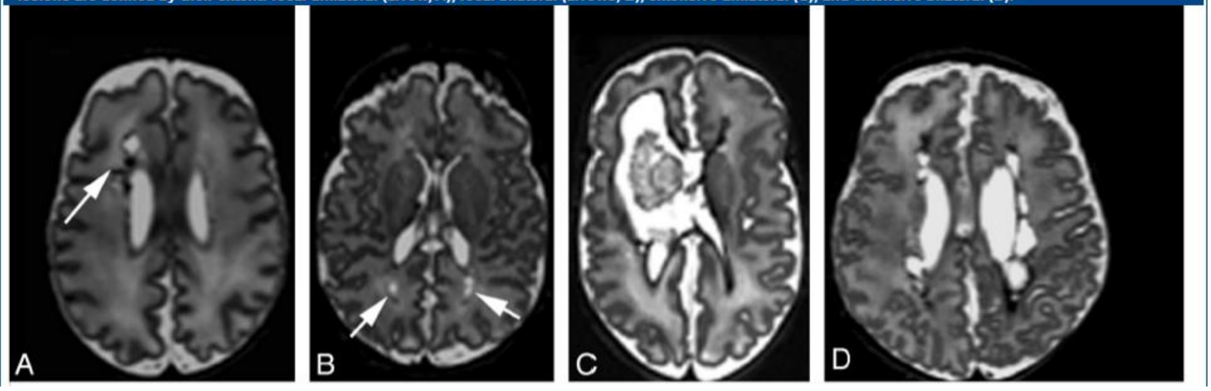

Focal signal abnormalities
0 = None;
1 = focal punctate;
2 = Extensive punctate;
3 = Linear

Help: Focal WM signal abnormalities (E–G). Focal WM signal abnormalities are classified as focal punctate (arrows, E), extensive punctate (arrows, F), or linear lesions corresponding to gliosis (arrows, G).

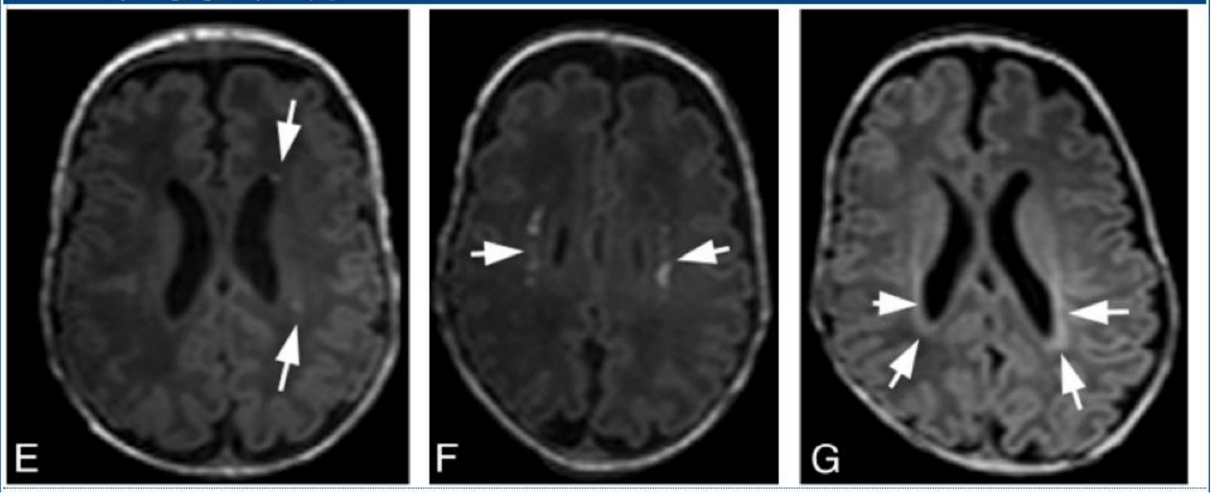

Delayed myelination
0= PLIC + corona radiata;
1 = Only PLIC;
2 = Minimal - no PLIC

Cortical Gray Matter

Signal abnormality
0 = None;
1 = focal punctate;
2 = Extensive punctate;
3 = Linear

Gyral maturation
0= Delay <2 weeks;
1 = 2 ≤ Delay < 4 weeks;
2 = Delay ≥ 4 week

Deep GM

Signal abnormality
0 = None;
1 = focal punctate;
2 = Extensive punctate;
3 = Linear

Cerebellum

Signal abnormality
0 = None;
1 = Punctate unilateral;
2 = Punctate unilateral;
3 = Extensive unilateral;
4 = Extensive bilateral

Help: Deep GM and cerebellar injuries are classified into 4 grades by their extent. Representative images of focal unilateral (arrow, H) or extensive unilateral (arrow, I) deep GM lesions, and images of focal bilateral (arrows, J) or focal extensive (arrow, K) cerebellar lesions are shown.

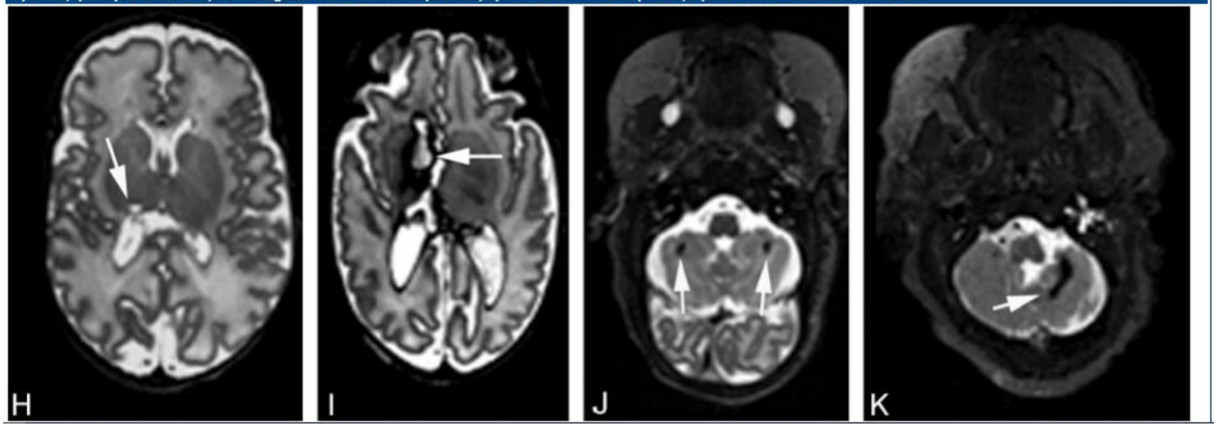

Regional measurement

Thinning of corpus callosum:

genu (mm)

body (mm)

splenium (mm)

Score Thinning of the corpus callosum
0 = None
1 = Partial (genu/body<1.3 mm or spelium<2.0mm)
2 = Global (genu/body < 1.3 mm and spelium<2.0mm)

Help: Callosal thickness is measured on a midsagittal view at 3 different regions: the genu, the midportion (body), and the splenium.

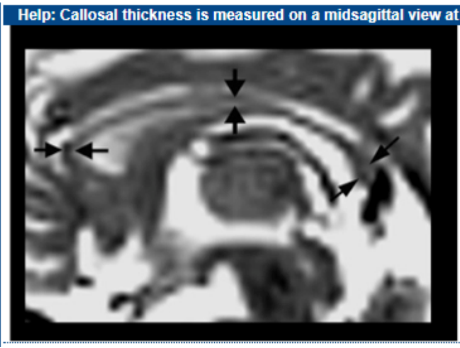

Dilated lateral ventricules

R-VD (mm)

L-D (mm)

Score dilated lateral ventricules

0 = Both sides VD < 7.5mm
1 = One side 7.5mm≤ VD≤ 10mm
2 = Both sides 7.5mm≤ VD≤ 10mm or one side VD≥10mm
3 = Both sides VD≥10mm

Help: C, Ventricular diameters (VDs) and transcerebellar diameter (TCD) are measured on a coronal view at the level of the ventricular atrium.

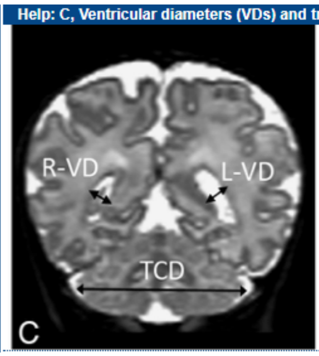

Cerebellum Volume reduction

TCD (mm)

Score volume reduction

0 = TCD ≥50mm
1 = 50 mm>TCD≥47mm
2 = 47 mm>TCD≥44mm
3 = 44mm

Reduction of WM volume

BPW (mm)

Score volume reduction

0 = BPW≥77mm
1 = 77mm>BPW≥72mm
2 = 72mm>BPW≥67mm
3 = 67mm>BPW

Extracerebral space

IHD (mm)

Score increased extracerebral space

0 = IHD <4 mm

1 = 4mm ≤ IHD <5 mm
2 = 5mm ≤ IHD <6 mm
3 = IHD ≥6mm

Help: A, Biparietal width (BPW) and interhemispheric distance (IHD) are measured on a single coronal section by use of the cochlea and basilar truncus as landmarks.

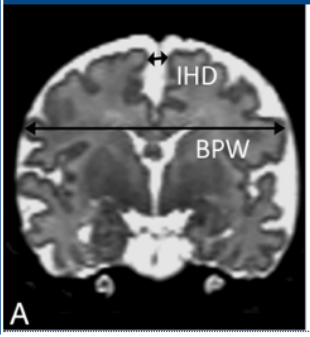

Deep GM volume reduction
DGMA mm2

0 = DGMA ≥ 950 mm2;
1 = 950 > DGMA ≥ 850 mm2;
2 = 850 > DGMA ≥ 750 mm2;
3 = 750 > DGMA

Help: D, The deep GM area (DGMA) is measured on a single axial section at the level at which the caudate heads, the lentiform nuclei, and the thalami are maximally visible.

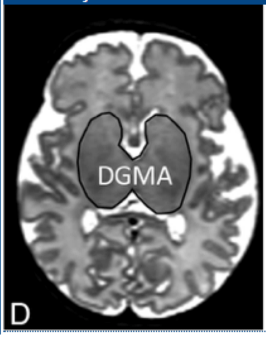

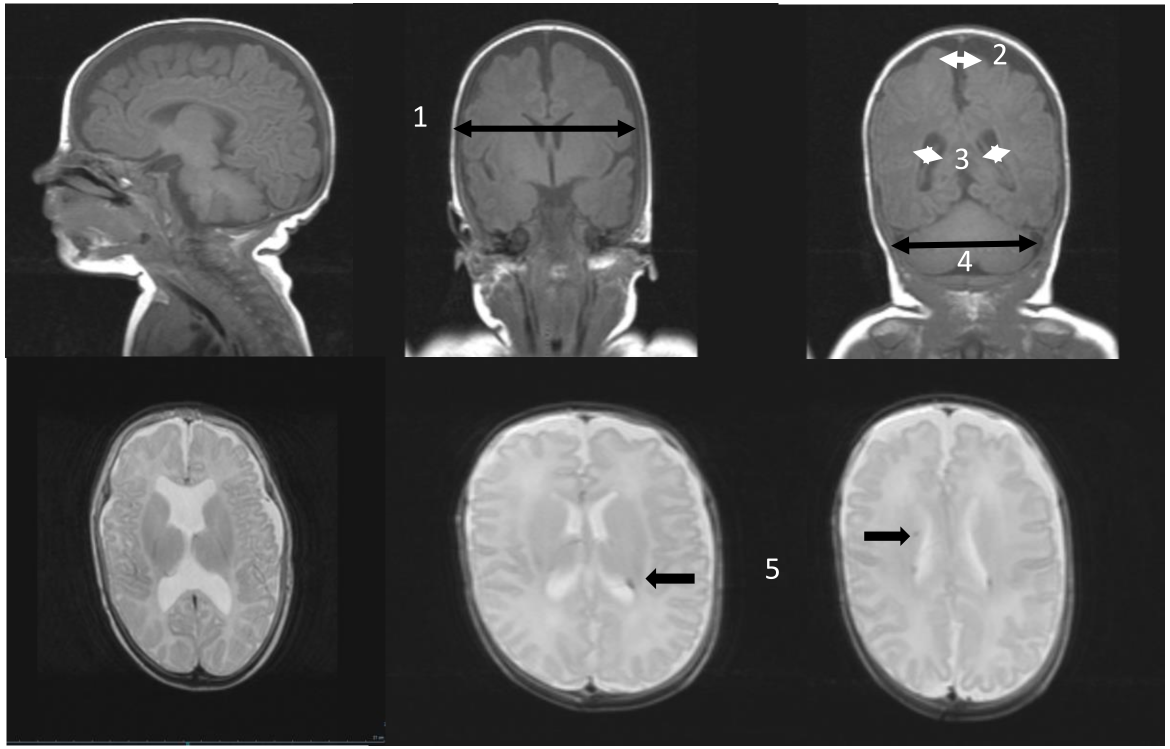

MRI at term equivalent age (42 GA) at an extremely preterm infants (25 GA, 630g): (1) shows the presence of periventricular white mater loss including moderate to severe anomaly of biparietal diameter (BPW 7mm), (2) an increase extracerebral space (IHD 3.7mm), (3) presence of lateral ventricular dilatation (R-VD 7.3mm, L-VD 5.5mm), (4) the presence of moderate to severe anomaly on the transcerebellar diameter (TCD 5.6mm), and (5) the presence of cystic lesions. At school age, this child had an ideomotor dyspraxia and a language delay.
